# The Real-world Therapeutic Analysis of First-line Immunotherapy in Chinese Patients with Drive Gene Positive for Advanced Non-Small Cell Lung Cancer

**DOI:** 10.7150/jca.77199

**Published:** 2023-04-01

**Authors:** Lei Liu, Fuxia Li, Jing Zhao, Xiaoli Zhuo, Jingjiang Lai, Jingliang Wang, Fengxian Jiang, Wei Xu, Fang Luan, Xiaoyan Lin, Shuping Yang, Guobin Fu

**Affiliations:** 1 Department of Oncology, Shandong Provincial Hospital Affiliated to Shandong First Medical University, Jinan, Shandong, China; 2 The Clinical Medical College, Shandong First Medical University (Shandong Academy of Medicine), Jinan, Shandong, China; 3 Department of Oncology, The Second Clinical Medical College, Shandong University of Traditional Chinese Medicine, Jinan, Shandong, China; 4 Department of Oncology, Shandong Provincial Hospital Cheeloo College of Medicine, Shandong University, Jinan, Shandong, China; 5 Department of Clinical Laboratory, Shandong Provincial Hospital Affiliated to Shandong First Medical University, Jinan, Shandong, China; 6 Department of Pathology, Shandong Provincial Hospital, Cheeloo College of Medicine, Shandong University, Jinan, Shandong, China; 7 Department of Pathology, Shandong Provincial Hospital Affiliated to Shandong First Medical University, Jinan, Shandong, China

**Keywords:** Non-small cell lung cancer, Driver Gene mutation, Immune checkpoint inhibitor, Survival analysis

## Abstract

**Background:** Immune checkpoint inhibitors (ICIs) are widely used for treating advanced non-small cell lung cancer (NSCLC). However, some studies indicate that patients with genetic mutations do not benefit from immunotherapy. Hence, this study explored the efficacy of anti-programmed death-1 (PD-1) and anti-programmed death-ligand 1 (PD-L1) antibodies in the first-line treatment of advanced NSCLC with driver gene mutations in real-world settings.

**Methods:** We retrospective analyzed patients with advanced NSCLC who treated with first-line anti-PD-1/PD-L1 antibodies at Shandong Provincial Hospital between May 2019 and October 2020. The patient's driver gene mutation status was identified using amplification refractory mutation system PCR (ARMS-PCR). The basic clinical characteristics, objective response rate (ORR), progression free survival (PFS), and other clinical data of patients were collected to evaluate the clinical efficacy and potential prognostic factors of treatment for patients with driver gene mutations.

**Results:** A total of 430 patients' information was counted during this period, finally, 89 patients with NSCLC were enrolled in the study. The main pathological subtype of patients was adenocarcinoma (62.9%). The overall mutation rate was 44.9% (n = 40) and included following mutations: *KRAS* (n = 20), *TP53* (n = 18), *EGFR* (n = 6), *BRAF* (n = 3), *Her-2* (n = 3), *MET* (n = 3), *ROS1* (n = 1), and *NRAS* (n = 1). The overall ORR was 44.30% and the disease control rate (DCR) was 82.23%. At the time of follow-up cut-off, the median PFS of all patients was 8.2 month. In NSCLC patients treated with ICI, median PFS was longer in mutation-negative patients than in mutation-positive patients (8.98 vs 7.07 months, P < 0.05). Survival benefit varied across mutational subgroups: KRAS patients could benefit from first-line immunotherapy (10.1 months, P < 0.05), patients with EGFR mutations have poor first-line immunotherapy outcomes, with a median PFS of only 3.0 months (P < 0.01), and patients with other mutation types having no significant difference in response from mutation-negative patients. In most mutation subgroups, immune combination therapy had longer PFS than immune monotherapy, and PD-L1 expression levels were positively correlated with clinical benefit in patients.

**Conclusion:** In the real world, patients with KRAS mutations benefit from first-line immunotherapy, immune-combination modalities are more effective, and immune efficacy is positively correlated with PD-L1 expression; Patients with other driver mutations (BRAF, NRAS, Her2, MET, ROS1) benefit similarly to mutation-negative patients in first-line immunotherapy, and immunotherapy is recommended for first-line therapy; Immunotherapy is worse effective in patients with EGFR mutations, immunotherapy is not recommended in first-line therapy even patients with high PD-L1 expression.

## Background

According to the 2019 American Cancer Report, lung cancer had the second highest incidence rate and the highest mortality rate among malignant tumors, in addition, it is the most common malignant tumor in China 1. Non-small cell lung cancer (NSCLC) is the most common type of lung cancer, accounting for 80% -85% of all lung cancer types. The positive proportion of driver gene in Chinese patients with advanced NSCLC is much higher than that in Europe and the United States 2, 3. The 5-year survival rate is only 19.7%, posing a serious threat to people's health 4. At present, the treatment of NSCLC is mainly determined by the driving gene. Compared with chemotherapy, the first-line TKI drug treatment for patients with EGFR mutation and ALK fusion significantly prolonged OS 5, 6. The first-line TKI drug treatment for NSCLC patients with driver gene mutation is the preferred treatment recommended by NCCN guidelines at present. For KRAS and other mutation types, targeted drugs have not been approved in China 7, and with the discovery of various new mutation sites and the emergence of complex drug resistance targets, how to improve the survival of patients with driver gene mutations has become a problem to be considered in clinical work.

The appearance of immune checkpoint inhibitors has greatly improved the prognosis of some lung cancer patients and provided a new direction for the clinical treatment of lung cancer. However, progress of research, clinicians found that the overall efficacy of first-line immunotherapy in NSCLC patients with partial driver gene mutations was poor, and the efficacy of EGFR/ALK mutation patients receiving ICI treatment was limited, even some patients experienced hyperprogression^8^, but there were still many case reports that such populations could benefit from immunotherapy. A mata analysis including five clinical trials showed that ICI treatment significantly prolonged NSCLC patients with wild-type EGFR (95% CI = 0.60 - 0.75; P < 0.001), but ICI treatment did not prolong OS in the EGFR mutation subgroup (95% CI = 0.80 - 1.53; P = 0.540) 9. China's special proportion of patients with driver gene mutations and the unreachability of some mutation targeted drugs make it important to clarify the correlation between the existence of driver gene and the efficacy of immunotherapy to guide Chinese clinicians to use drugs.

Hence, in this study, we retrospectively observed patients with advanced NSCLC containing driver gene mutations to observe the prognosis of patients receiving first-line ICIs in the real world. In addition, we aimed to clarify the relationship between the presence of driver gene mutations and immune efficacy to provide recommendations for immunotherapy in these patients.

## Patients and Methods

### Study Population

We selected patients with NSCLC treated with anti-PD-1/PD-L1 antibodies at Shandong Provincial Hospital between May 2019 and October 2020. The inclusion criteria were as follows: (1) NSCLC diagnosed by histopathology, and at least one measurable lesion; (2) single agent ICI therapy with commercial anti-PD1/PD-L1 antibodies in our hospital; (3) clear genetic test results; (4) local response assessment according to RECIST1.1 criteria.

The exclusion criteria were as follows: (1) Surgical treatment during treatment; (2) pathological type conversion during treatment; (3) failure to complete the efficacy evaluation or loss to follow-up; (4) combined with other malignant tumors.

### PD-L1 analysis

89 individuals with advanced NSCLC underwent PD-L1 immunohistochemistry utilizing paraffin-embedded tissue slices. The 3-mm-thick, hematoxylin and eosin-stained segment that included the tumor cells was examined. For measurement of PD-L1 expression, the specimen had to include at least 100 tumor cells that were still viable. All 89 patient specimens underwent a commercial PD-L1 IHC assay, SP263 (Spring Bioscience, Ventana; Tucson, AZ). Using the VENTANA Bench-Mark ULTRA system, sections were stained with anti-PD-L1 SP263 rabbit monoclonal antibody (dilution 1:100). Tumor cells showing membranous staining for PD-L1 were considered as the positive cells. The proportion of tumor cells (tumor proportion score, TPS) with PD-L1 expression was estimated as the percentage of total tumor cells. All stained slides were assessed by two experienced pathologists for PD-L1 membrane staining. Sections from the human placenta were used as positive controls for PD-L1 expression.

### Molecular diagnostics

We performed genetic testing using ARMS-PCR. It uses known point mutations to design primers, complements the clips of unmutated and mutated templates with their 3-terminal bases, and achieves the purpose of distinguishing templates with a certain point mutation from normal templates. The tissue specimens used were obtained from biopsy aspirated NSCLC formalin-fixed paraffin-embedded (FFPE) specimens. The ARMS-PCR technology was provided by the Department of Pathology, Provincial Hospital Affiliated with Shandong First Medical University.

### Ethical considerations

The study was conducted in accordance with the Declaration of Helsinki and approved by the Biomedical Research Ethics Committee of Shandong Provincial Hospital (SWYX: No. 2022-052). The participating centers acquired the consent of patients and institutional approval. All contributors were trained in Good Clinical Practice. The study was not funded by industry.

### Data collection

Clinical characteristics of the patients with NSCLC at the time of initial immunotherapy were obtained from the electronic medical records and included the following: age, gender, smoking status, cancer stage, number and site of metastases, mutation status of 10 common clinical genes, namely, *EGFR*, *KRAS*, *TP53*, *BRAF*, *NRAS*, *Her2*, *MET*, *RET*, *ALK*, *ROS1*, number of lines of treatment before ICI, and clinical response to ICI treatment. Histological subtypes were determined according to the 2004 World Health Organization classification. The tumor stage was identified based on the American Joint Committee on Cancer's 7th edition lung cancer staging system. Patients were tracked from the initial administration of immunotherapy until death or the last follow-up date.

### Treatment plan

According to the treatment modality, patients were divided into Immune monotherapy and Combination immunotherapy. The ICIs included Camrelizumab, Durvalumab, Pembrolizumab, Sintilimab, and Tislelizumab. The chemotherapy drugs included Albumin-bound paclitaxel, Cisplatin, Gemcitabine, Pemetrexed. The antiangiogenic drugs included Bevacizumab.

### Efficacy evaluation

We performed computed tomography (CT), cranial magnetic resonance imaging (MRI), and single-photon emission computed tomography (SPECT) of the whole-body skeleton to assess baseline levels in all study subjects before starting regular immunotherapy, and efficacy was evaluated after every two cycles of treatment. Efficacy evaluation was divided into complete response (CR), partial response (PR), stable disease (SD), and progressive disease (PD) according to RECIST1.1. The primary objective was to determine the progression-free survival (PFS) of patients treated with anti-PD-1/PD-L1 antibodies (ICIs) in the mutant group and negative group. Secondary objectives were to determine the ORR for each molecular subgroup, analyze the outcome of patients according to the line of treatment, and analyze PD-L1 expression. PFS was calculated from the beginning of anti-PD-1 treatment to date of progression of disease or death. ORR is the sum of the proportion of complete response plus partial response.

### Statistical analyses

For data description, mean values and standard deviations were used. Categorical data were analyzed by χ2 test. Estimation of PFS was carried out using the Kaplan-Meier method with the log-rank test. The level of statistical significance was determined at P = 0.05. All statistical analyses were performed using SPSS 26 statistical software (IBM Corp., Armonk, NY, USA).

## Results

### Clinicopathological characteristics of patients

**Figure 1:**
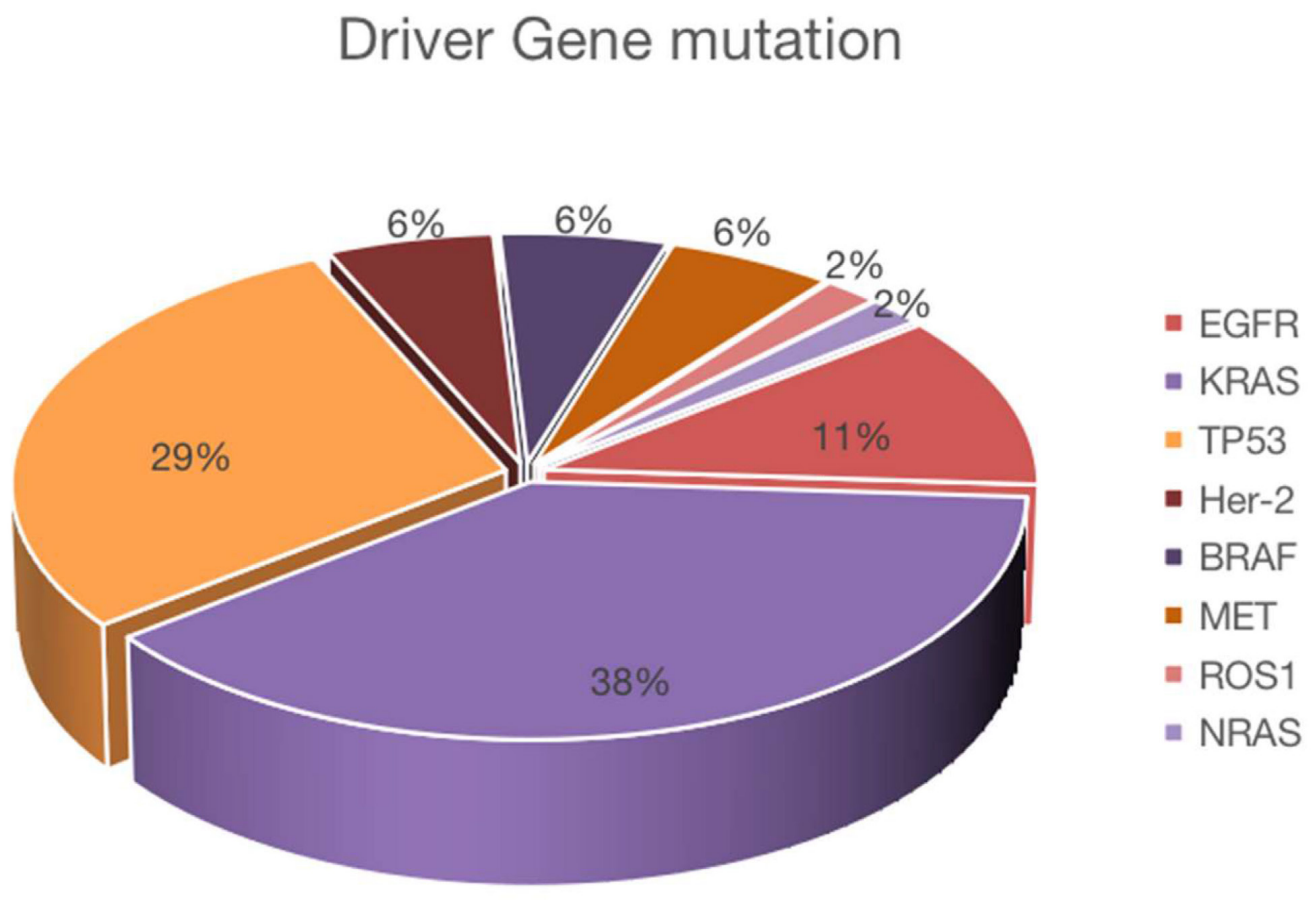
Proportion of mutations in driver gene positive NSCLC. Forty of 89 NSCLC patients were positive for driver mutations, and the number of each mutation was as follows: KRAS (n = 20), TP53 (n = 18), EGFR (n = 6), BRAF (n = 3), Her-2 (n = 3), MET (n = 3), ROS1 (n = 1), and NRAS (n = 1).

From May 2019 to October 2020, we counted a total of 430 patients receiving immunotherapy, and after excluding non-NSCLC, not receiving first-line immunotherapy, lack of exact mutation type, and missing survival data, a total of 89 patients with NSCLC receiving first-line immunotherapy were finally included. Among the included patients, 85.4% were males, 62.9% had adenocarcinoma, 60.7% were at stage IV, 78.7% were former smokers, and 44.9% had driver gene mutations. For patients receiving first-line immunotherapy, the driver gene mutations are shown in Figure 1, the clinical information in Table 1, and the univariate and multivariate analyses in Table 2. Because of the low mutation rate, except for KRAS, TP53, and EGFR, other mutation subtypes we performed a pooled analysis and named them as uncommon mutations.

### Treatment characteristics

**Figure 2:**
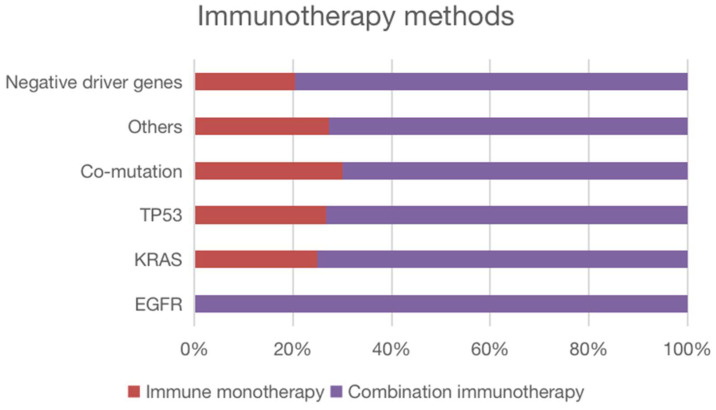
Immunotherapy of patients in each mutation subgroup and mutation-negative group. All patients with EGFR mutations were treated with immune combination therapy; one quarter of patients with KRAS mutations were treated with immune monotherapy; four patients with TP53 mutations were treated with immune monotherapy; seven patients with Co-mutation were treated with immune combination therapy; three patients in the Other groups were treated with immune monotherapy; among patients with negative driver mutations, 10 patients were treated with immune monotherapy and the rest were treated with immune combination therapy.

We observed that 98% of the patients received anti-PD1-antibodies (Camrelizumab n = 43, Pembrolizumab n = 12, Sintilimab n = 19, and Tislelizumab n = 13), while only 2% received anti-PD-L1-antibodies (Durvalumab n = 2). Patients with NSCLC receiving first-line immunotherapy have multiple treatment modalities, as shown in Figure 2.

### PD-L1 expression

**Figure 3:**
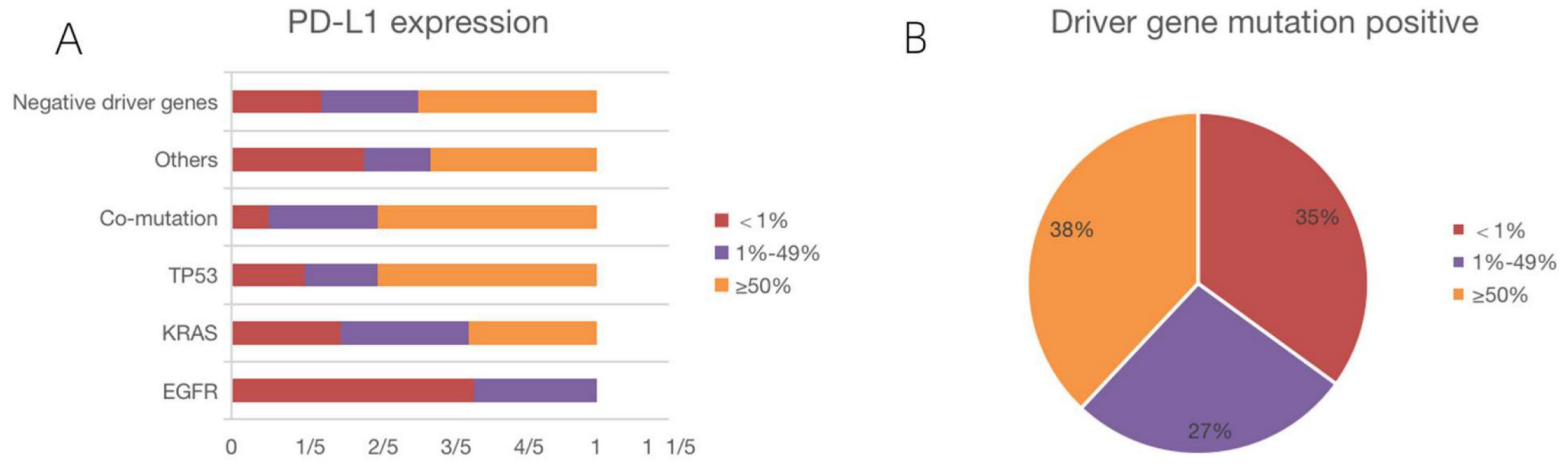
PD-L1 expression of patients in each mutation subgroup. PD-L1 was low expressed in 2 patients with EGFR mutations, and the remaining 4 patients expressed negative; among KRAS mutations, 7 patients were low expressed, 7 patients were high expressed, and the remaining 6 patients were negative; among TP53 mutations, 3 patients were low expressed, 9 patients were high expressed, and the remaining 3 patients were negative; among Co-mutations, 3 patients were low expressed, 6 patients were high and the remaining 1 patient was negative; among Others, 2 patients had low expression, 5 patients had high expression, and the remaining 4 patients were negative; among all patients with negative driver mutations, 13 patients had low expression, 24 patients had high expression, and the remaining 12 patients were negative.

**Figure 4:**
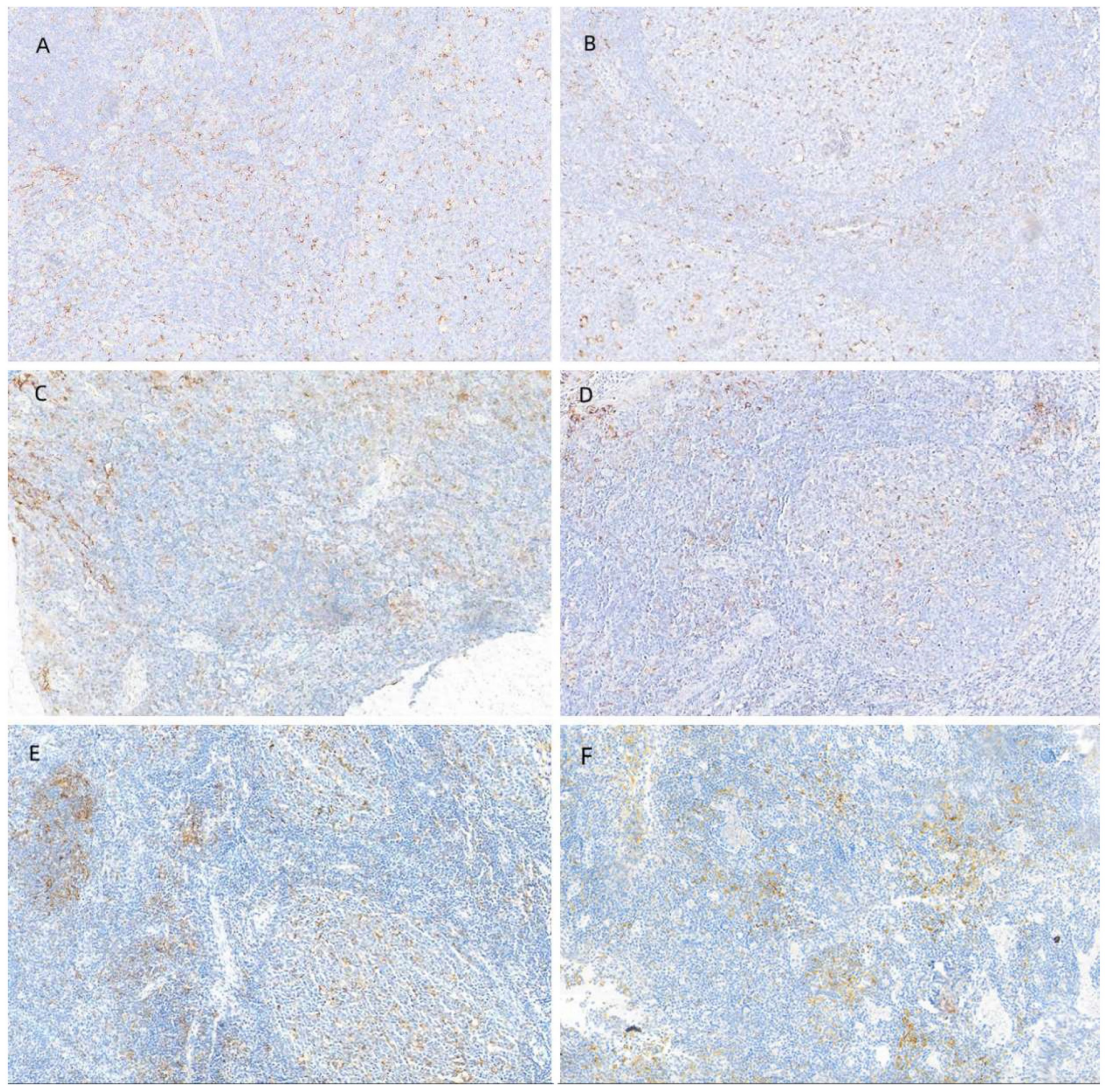
IHC images of patients with different PD-L1 expression intensities. (A, B) Negative (<1%) PD-L1, (C, D) low (1% - 49%) PD-L1 expression, and (E, F) high (>50%) PD-L1 expression.

We counted PD-L1 expression in all patients. Figure 3A depicts PD-L1 expression in each subgroup, and Figure 3B illustrates PD-L1 expression in driver gene mutation-positive patients. The median number of positive cells was 49%. Using a 1% cut-off, 70.0% were positive, whereas using a 50% cut-off, 42.7% of the tumors were positive. We also collected IHC images of patients with different PD-L1 expression intensities, and Figure 4 shows the IHC results of a few patients.

### Clinical outcomes

#### Progression-free survival

**Figure 5:**
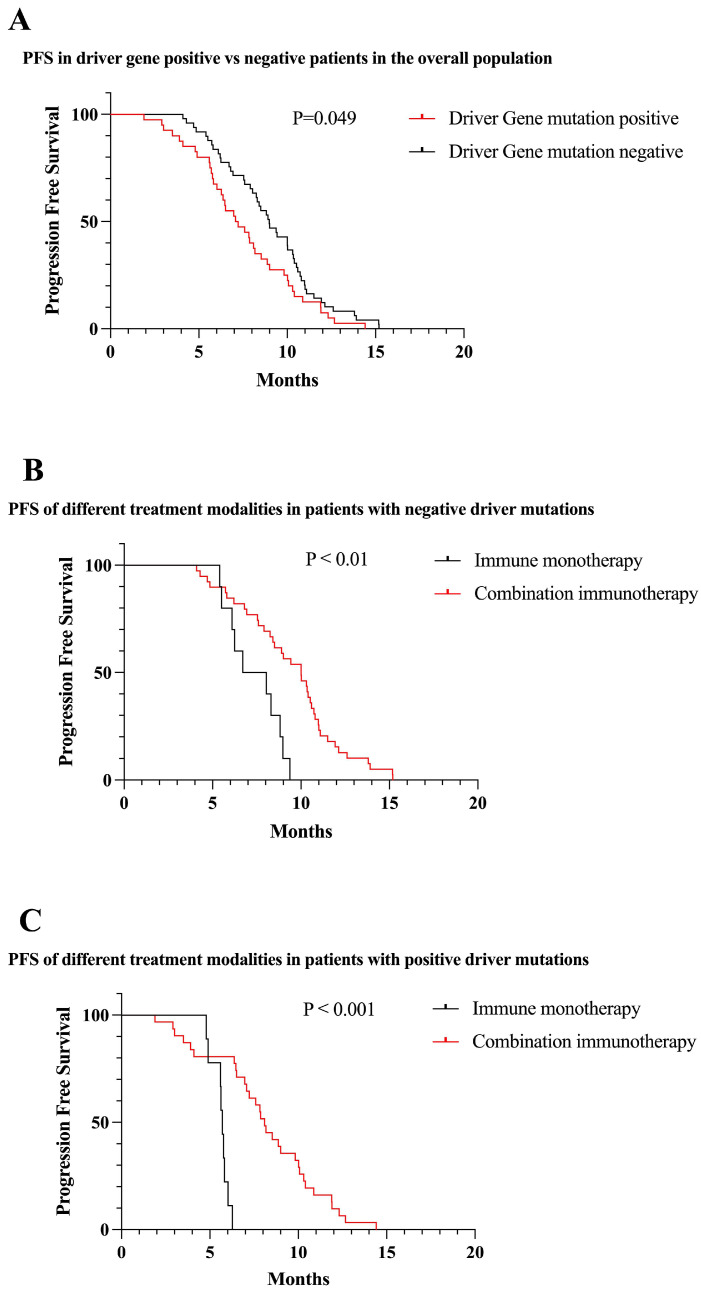
Survival curves of the total patients enrolled in the different conditions. (A) PFS of patients with positive driver gene mutations vs negative patients (mPFS: 7.07 vs 9.00 months, P = 0.049). (B) First-line immune monotherapy in driver gene mutation-negative patients had inferior mPFS compared with immune combination therapy (mPFS: 7.34 vs 9.44 months, P < 0.01). (C) First-line immune monotherapy in driver gene mutation-positive patients had inferior mPFS compared with immune combination therapy (mPFS: 5.61 vs 8.08 months, P < 0.001).

Among the included patients, 44.9% had driver gene mutations. Overall, among all patients included, PFS was lower in patients with positive driver mutations than in patients with negative mutations (7.07 vs 9.00 months, P = 0.049) (Figure 5A). According to the survival curve analysis, PFS was better with the immune combination therapy than with immune monotherapy, both in patients with positive driver gene mutations and in patients with negative driver gene mutations (P < 0.01) (Figure 5B,C).

In the univariate analysis, PFS significantly correlated with the histologic type. Meanwhile, in multivariate analysis, PFS significantly correlated with treatment mode. However, PFS did not correlate with gender, age and stage.

#### Molecular subgroup analyses

##### EGFR mutations

**Figure 6:**
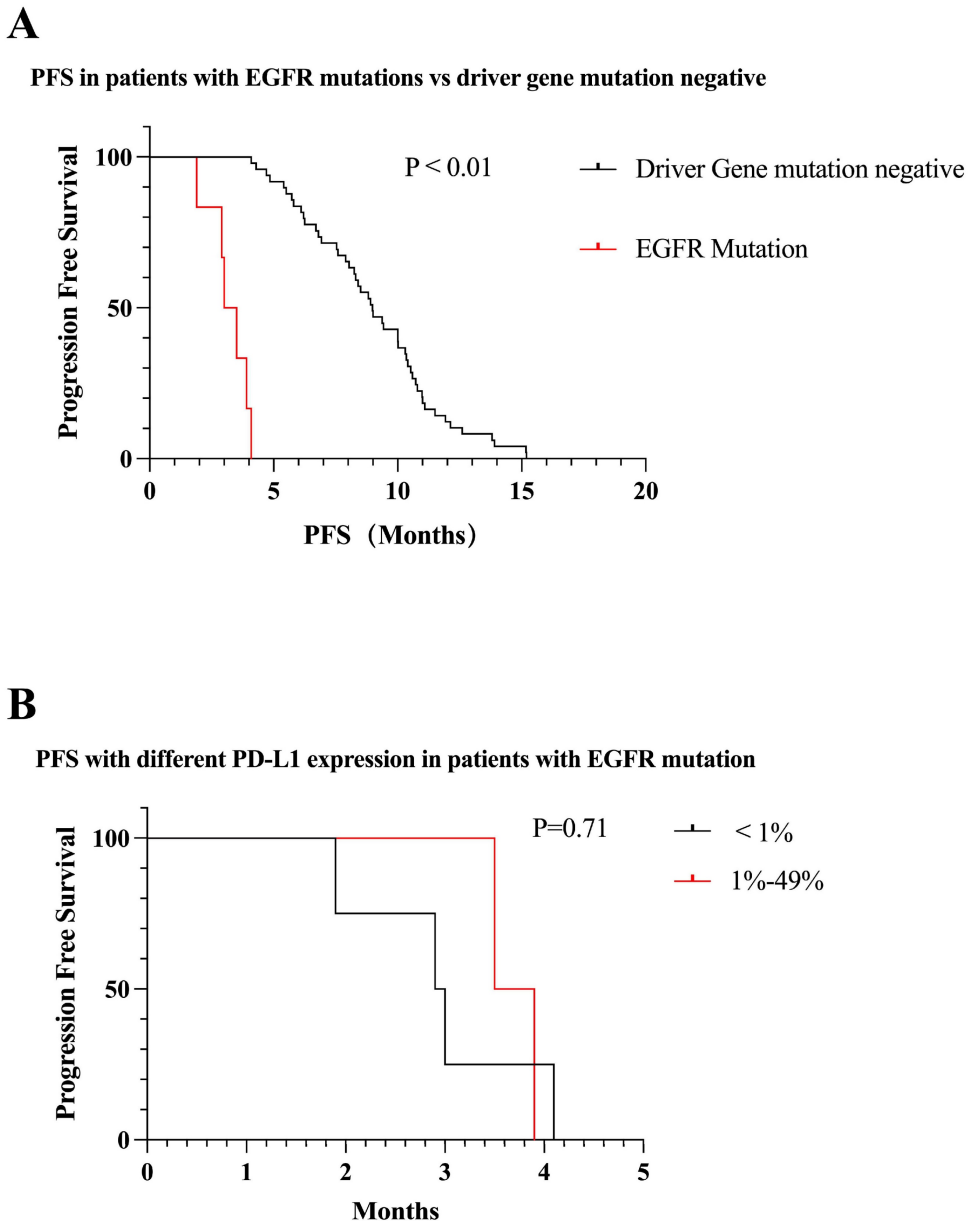
Survival curves for patients with EGFR mutations. (A) Patients with EGFR mutations receiving first-line immunotherapy have much lower mPFS than mutation-negative patients (mPFS: 3.0 vs 9.0 months, P < 0.01). (B) mPFS in patients with positive PD-L1 expression in patients with EGFR mutations was not statistically different from that in patients with negative PD-L1 expression (mPFS: 2.9 vs 3.5 months, P = 0.71).

We identified six patients with *EGFR* mutations. The PFS was significantly less in the *EGFR* mutation-positive patients than in the mutation-negative patients (3.0 vs 9.0 months, P < 0.01) (Figure 6A). Among all patients with *EGFR* mutations, 2/3 of patients were negative for PD-L1 expression. PFS was lower in patients with negative PD-L1 expression compared to those with positive PD-L1 expression (2.9 vs 3.5 months, P = 0.71) (Figure 6B). All *EGFR* patients were treated with immune combination therapy with a mean PFS of 3.2 months.

##### KRAS mutations

**Figure 7:**
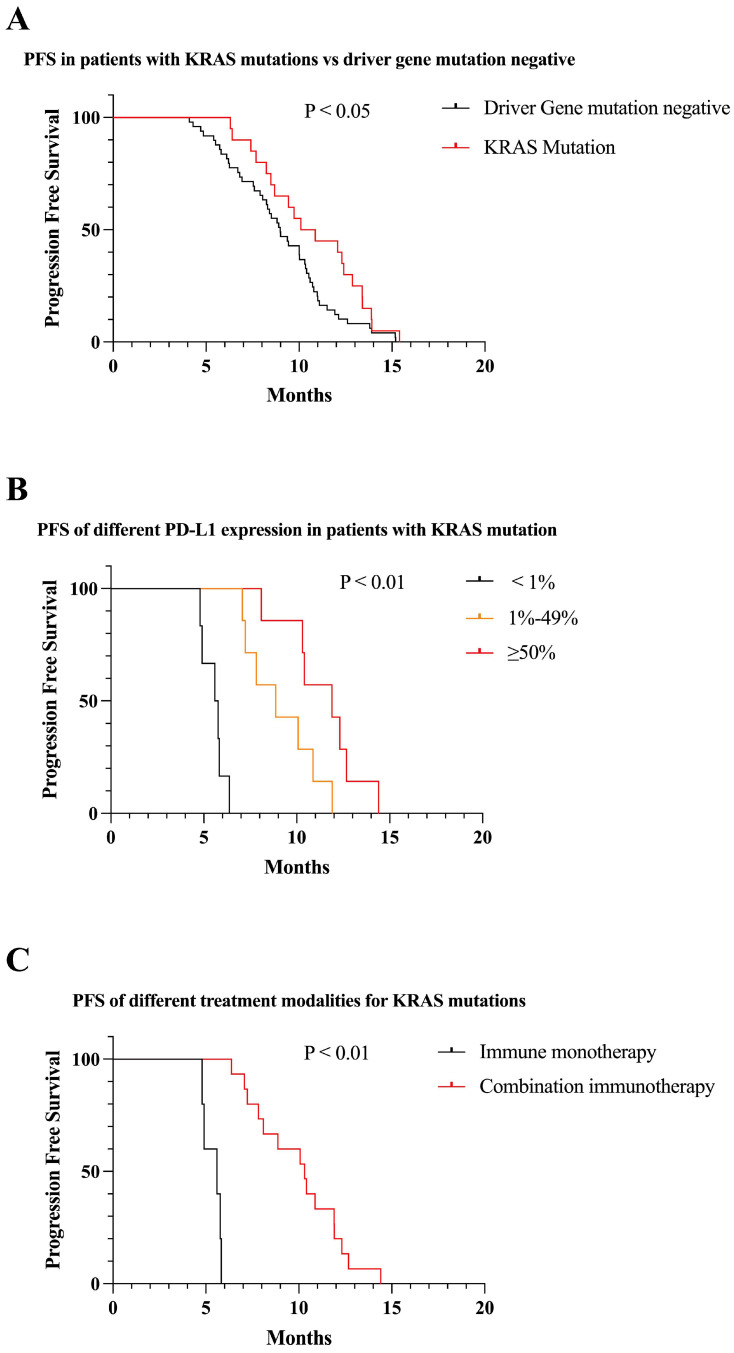
Survival curves for patients with KRAS mutations. (A) KRAS mutations patients received first-line immunotherapy with higher mPFS than mutation negative patients (mPFS: 10.1 vs 9.0 months, P < 0.05). (B) In patients with KRAS mutations, PFS gradually increased as PD-L1 expression increased (mPFS: 7.4 vs 10.9 vs 13.4 months, P < 0.01). (C) First-line combination immunotherapy showed longer PFS than immune monotherapy in KRAS mutations patients (mPFS: 10.3 vs 5.6, P < 0.01).

We identified 20 patients with *KRAS* mutations. *KRAS* mutation patients had significantly higher PFS than mutation-negative patients (10.1 vs 9.0 months, P < 0.05) (Figure 7A). Among patients with *KRAS* mutations, patients who were positive for PD-L1 expression had longer PFS than those who were negative, and PFS increased with increasing PD-L1 expression (7.4 vs 10.9 vs 13.4 months, P < 0.01) (Figure 7B). One quarter of patients in *KRAS* mutations received immune monotherapy, which had poorer efficacy and shorter PFS compared to immune combination therapy (5.6 vs 10.3, P < 0.01) (Figure 7C).

##### TP53 mutations

**Figure 8:**
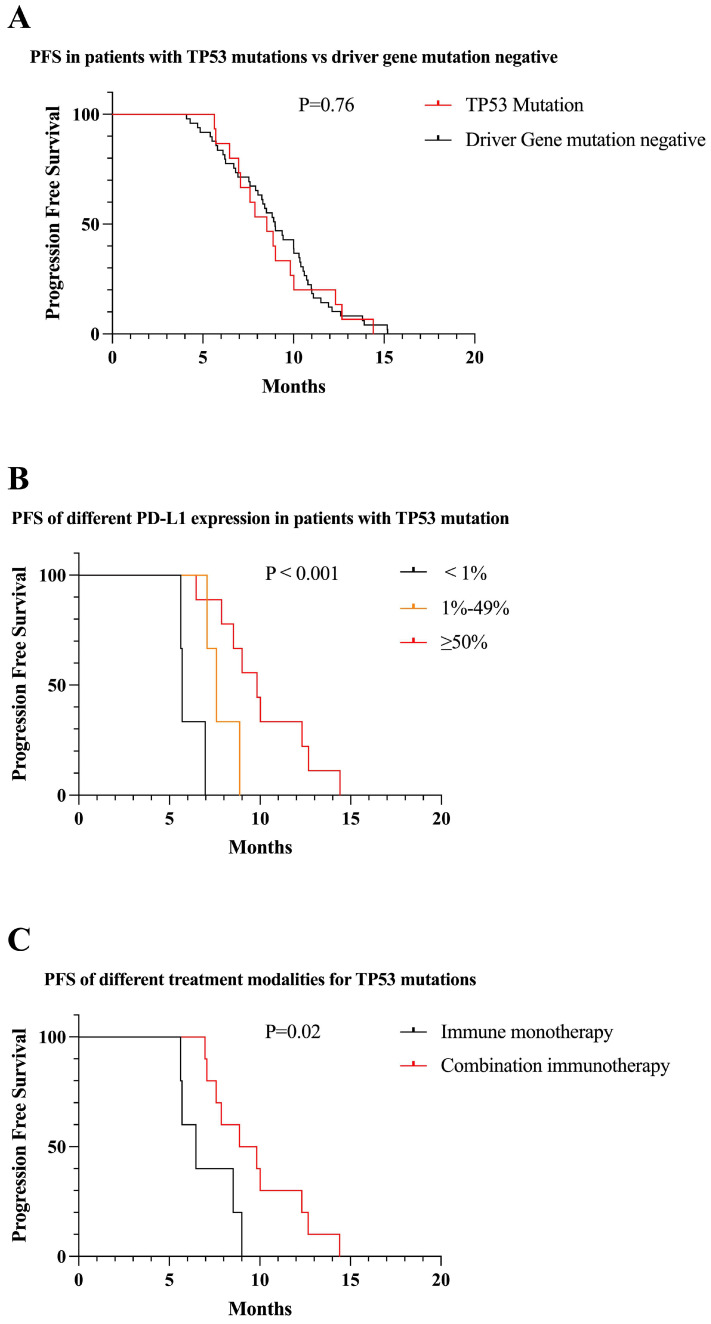
Survival curves for patients with TP53 mutations. (A) The mPFS of patients with TP53 mutations receiving first-line immunotherapy was similar to mutation-negative patients (mPFS: 8.8 vs 9.0 months, P = 0.76). (B) In patients with TP53 mutations, PFS gradually increased as PD-L1 expression increased (mPFS: 6.1 vs 7.8 vs 10.1 months, P < 0.001). (C) First-line combination immunotherapy showed longer PFS than immune monotherapy in TP53 mutations patients (mPFS: 9.8 vs 7.1, P = 0.02).

We identified 15 patients with *TP53* mutations. There was no significant difference in PFS between TP53 mutations and driver gene mutation negative patients receiving first-line immunotherapy (8.8 vs 9.0 months, P = 0.76) (Figure 8A). Among patients with *TP53* mutations, patients who were positive for PD-L1 expression had longer PFS than those who were negative, and PFS increased with increasing PD-L1 expression (6.1 vs 7.8 vs 10.1 months, P < 0.001) (Figure 8B). Less than one-third of patients in *TP53* mutations received immune monotherapy, which had poorer efficacy and shorter PFS compared to immune combination therapy (7.1 vs 9.8, P = 0.02) (Figure 8C).

##### Uncommon mutations

**Figure 9:**
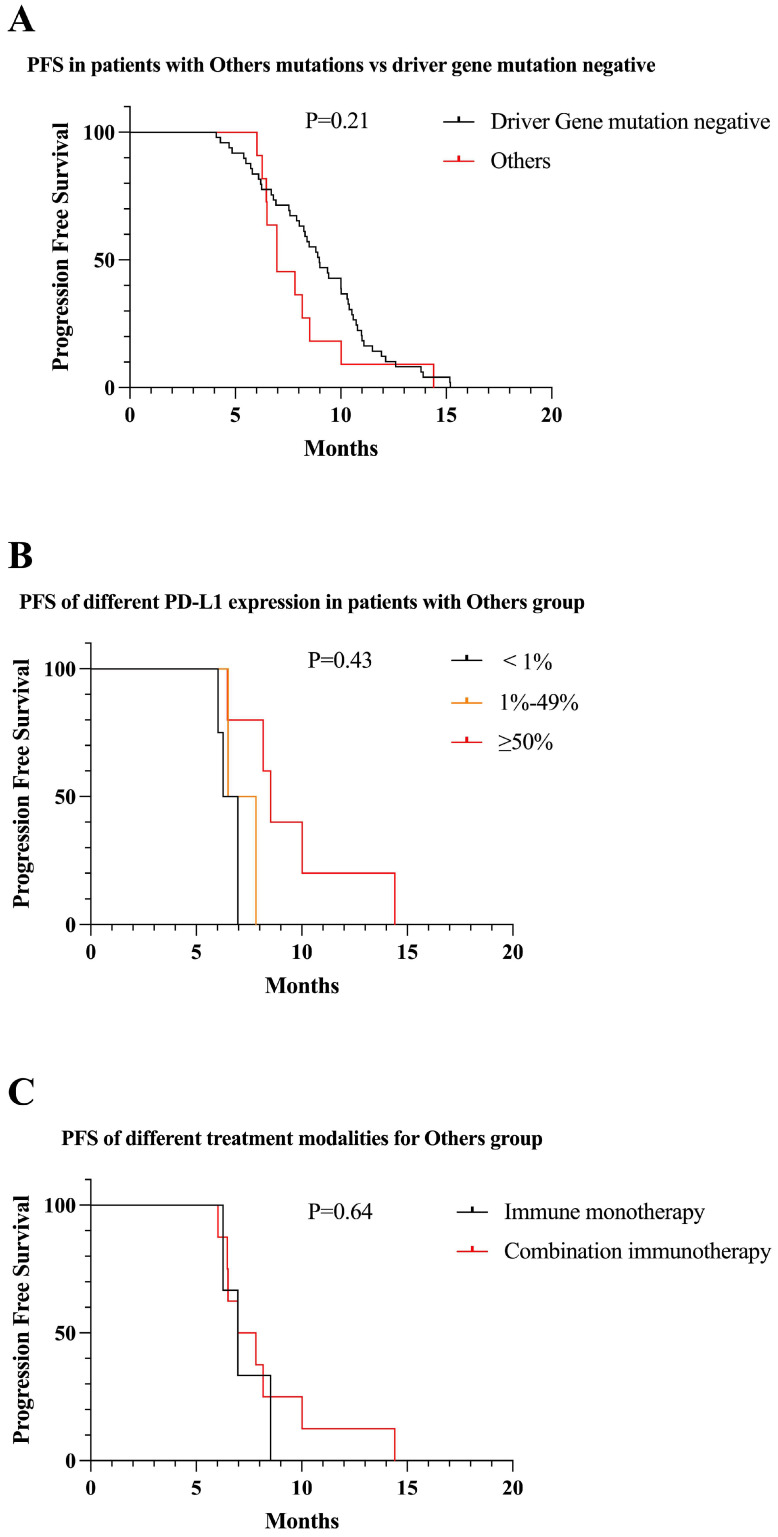
Survival curves for patients with “uncommon mutations” group. (A) The mPFS of patients with uncommon mutations receiving first-line immunotherapy was similar to mutation-negative patients (mPFS: 8.0 vs 9.0 months, P = 0.21). (B) In “uncommon mutations” group, PFS gradually increased as PD-L1 expression increased (mPFS: 6.5 vs 7.1 vs 9.5 months, P = 0.43). (C) Patients in “uncommon mutations” group, PFS with first-line combined immunotherapy was not statistically different from immune monotherapy (mPFS: 8.2 vs 7.2, P = 0.64).

Eleven patients were classified in “uncommon mutations” group, including BRAF, NRAS, Her2, MET, ALK, ROS1. There was no significant difference in PFS between uncommon mutations and driver gene mutation negative patients receiving first-line immunotherapy (8.0 vs 9.0 months, P = 0.21) (Figure 9A). Among patients with uncommon mutations, patients who were positive for PD-L1 expression had longer PFS than those who were negative (6.5 vs 7.1 vs 9.5 months, P = 0.43) (Figure 9B). Three patients in uncommon mutations received immune monotherapy, which did not reflect a statistical difference compared to immune combination therapy (7.2 vs 8.2, P = 0.64) (Figure 9C).

##### Co-mutation

**Figure 10:**
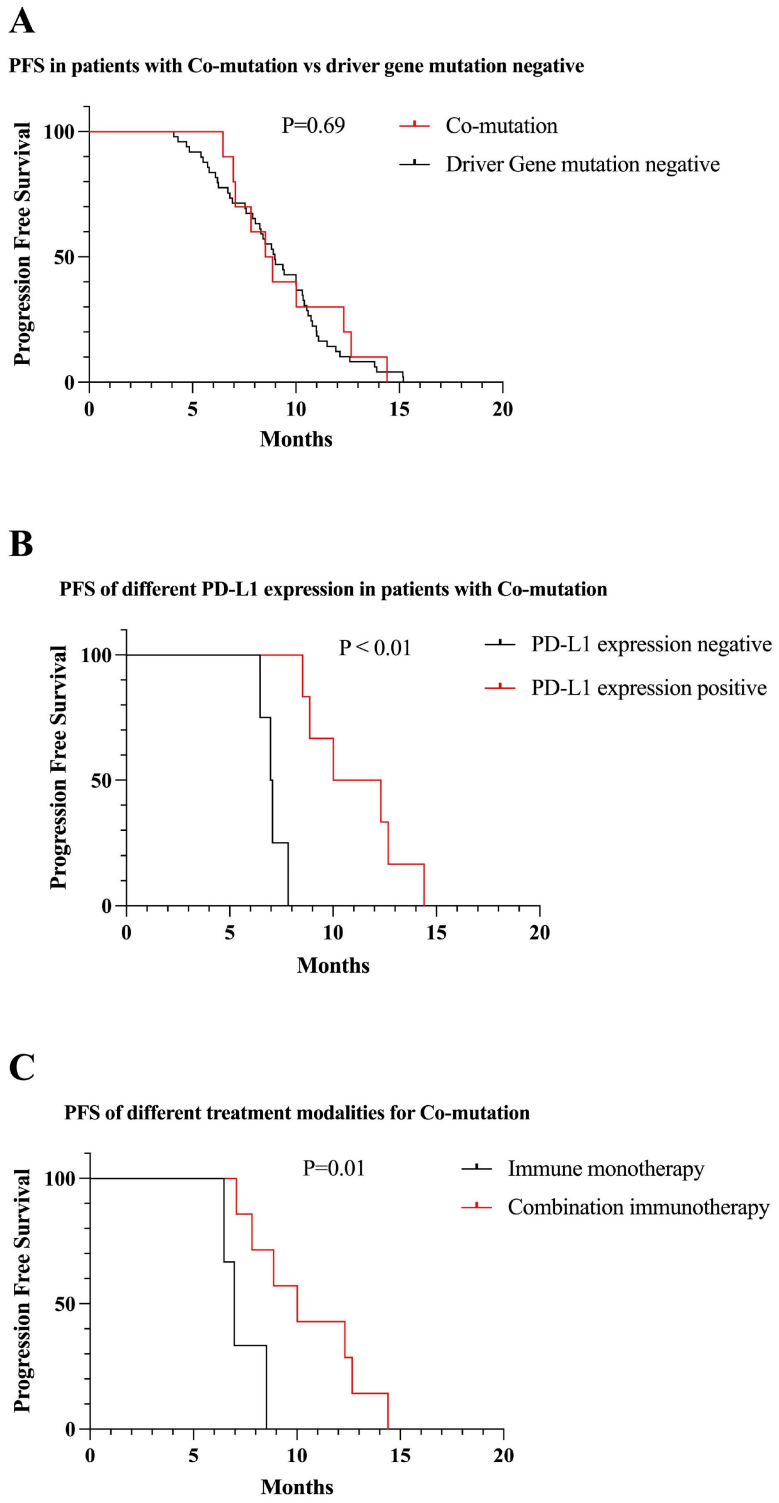
Survival curves for patients with Co-mutation. (A) The mPFS of patients with Co-mutation receiving first-line immunotherapy was similar to mutation-negative patients (mPFS: 9.5 vs 9.0 months, P = 0.69). (B) In Co-mutation patients, PD-L1 expression positive patients had longer PFS than negative patients receiving first-line immunotherapy (mPFS: 11.1 vs 7.1 months, P < 0.01). (C) First-line combination immunotherapy showed longer PFS than immune monotherapy in Co-mutation patients (mPFS: 10.5 vs 7.3, P = 0.01).

We identified 10 patients with *Co-mutation*. There was no significant difference in PFS between *Co-mutation* and driver gene mutation negative patients receiving first-line immunotherapy (9.5 vs 9.0 months, P = 0.69) (Figure 10A). Among patients with *Co-mutation*, patients who were positive for PD-L1 expression had longer PFS than those who were negative (7.1 vs 11.1 months, P < 0.01) (Figure 10B). One-third of patients in *Co-mutation* received immune monotherapy, which had poorer efficacy and shorter PFS compared to immune combination therapy (7.3 vs 10.5, P = 0.01) (Figure 10C).

## Discussion

In recent years, ICIs have brought a major breakthrough in the treatment of patients with advanced driver gene-negative NSCLC, and the 5-year survival rate of patients has increased from 5% 1 in the chemotherapy era to 13.4%-23.2% 10, 11, and ICIs have become the standard of care for advanced NSCLC. However, patients with driver gene-positive NSCLC represented by EGFR and ALK have been considered unable to benefit from immunotherapy 12, and most clinical trials on immune checkpoint inhibitors have excluded such patients, and we do not know the efficacy of immunotherapy for patients with driver gene mutations. Here, we included real-world patients with driver mutation-positive NSCLC who received immunotherapy in the first line to explore the trend of immunotherapy benefit in this population.

We retrospectively analyzed the efficacy and clinical factors associated with prognosis in a longitudinal cohort of real-world patients with advanced NSCLC treated with ICIs. In all, we included 89 patients with advanced NSCLC who received first-line immunotherapy and had an overall mutation rate of 44.9%. According to the follow-up results, in general, mutation-negative NSCLC patients benefit more from first-line immunotherapy than mutation-positive NSCLC patients, except in KRAS mutated patients. In each of the subgroups classified, the modality of immune combination therapy was higher than that of immune monotherapy PFS, and PD-L1 expression levels were positively correlated with patient PFS.

Numerous retrospective studies have assessed the efficacy of ICIs in patients with NSCLC containing driver gene mutations 13-15. Mazieres et al. 16 examined 551 patients with NSCLC containing mutations in the driver genes, including *KRAS*, *EGFR*, *ERBB2*, *ALK*, *ROS-1*, *BRAF*, and *RET*, and 94.6% of them received ICIs following TKI or chemotherapy progression. The data suggested that patients with NSCLC containing a driver gene mutation had relatively fewer benefits from ICIs. The median PFS and OS were 2.8 months and 13.3 months, respectively, and the median duration of follow-up was 3.2 months. Patients with KRAS mutation reaped the most benefits, while those with ALK mutation did not benefit in terms of survival. Our conclusions are consistent with previous studies: among the six EGFR mutant patients included, the median PFS was only 3.0 months, which is a significant gap with driver gene mutation-negative patients, suggesting that EGFR mutant patients have limited benefit from first-line immunotherapy. KRAS mutation, as a mutation type without targeted drugs in China, has been shown to be effective in first-line immunotherapy, with a median PFS of 10.1 months, becoming the only mutation type in this study that can benefit from immunotherapy. For other types of mutations, no differences in efficacy have been observed compared with patients without driver mutations, and therefore, ICIs may be considered for first-line treatment in patients with mutations who do not have targeted drug therapy.

Mutant lung cancer cells are associated with low PD-L1 expression, and this may be the prime reason patients with driver gene mutations benefit less from immunotherapy 17. A study including 54 *EGFR* mutation samples identified only 9 samples with PD-L1 expression > 5% accounted for 9.3%. An analysis of PD-L1 expression levels in 871 Chinese NSCLC samples showed that patients with EGFR mutations had slightly lower PD-L1 positivity (43.8% and 49.8%, respectively), while higher PD-L1 expression (TPS ≥ 50%) was significantly lower (14.3% and 27.4%, respectively) compared with EGFR wild-type patients 18. In addition, comprehensive analysis of PD-L1 and CD8^+^ TILs in 255 Chinese NSCLC samples showed that the proportion of PD-L1^+^ and CD8^+^ TILs double positive in TME was decreased, while the proportion of PD-L1^-^ and CD8^-^ TILs double negative was increased in EGFR mutation group compared with EGFR wild-type group patients (P < 0.01) 19. This plausibly explains the poor efficacy of ICIs in most real-world patients with driver mutation-positive NSCLC. Up to 2/3 of our enrolled patients with EGFR mutations were negative for PD-L1 expression, which may also explain the poor immune efficacy in this population.

In contrast, clinical samples showed higher expression of TMB, PD-L1, and TILs in KRAS mutant patients 20. TMB testing of clinical samples from 4017 driver gene-positive NSCLC patients showed higher TMB levels in KRAS mutant patients compared to other driver gene-positive patients (median 7.8 mut/Mb, n = 2240), suggesting higher immunogenicity of KRAS mutant tumors 20. A meta-analysis of 23 studies analyzed tissue samples from 5326 patients showed that patients with KRAS mutations had higher PD-L1 positivity than KRAS wild-type patients (P < 0.01) 21. The above results suggest that KRAS mutations are associated with high tumor immunogenicity and inflammatory microenvironment, and patients can potentially benefit from ICIs. In our study, the positive rate of PD-L1 expression in KRAS mutant patients was as high as 70%, which had a significant immunotherapeutic benefit compared with mutation-negative NSCLC patients, and PFS also gradually prolonged with increasing PD-L1 expression levels.

Immunotherapy modalities also play an important role in the survival benefit of patients. Basic studies have shown that chemotherapy is able to induce immunogenic cell death and promote tumor antigen release 22, 23. Bevacizumab promotes DC maturation and promotes more efficient T cell priming and activation, while it induces normalization of tumor vasculature, thereby promoting effector lymphocyte infiltration into tumor tissue 24. Bevacizumab can reduce the activity of myeloid-derived suppressor cells and Treg cells, remodel TME, and restore anti-tumor immune function through T cell-mediated tumor cell killing 25. Therefore, immune combination therapy modalities theoretically lead to better clinical benefit for patients. The KEYNOTE-189 study 26 compared the efficacy and safety of pembrolizumab combined with pemetrexed and platinum versus placebo combined with pemetrexed and platinum in treatment-naïve EGFR or ALK-negative metastatic non-squamous NSCLC patients, with KRAS mutations in 89 (31%) patients, and pembrolizumab combined with platinum-based doublet chemotherapy showed more PFS benefit compared with platinum-based doublet chemotherapy (mPFS 9.0 and 5.0 months, HR = 0.47, 95% CI: 0.29 to 0.77), with a similar trend of OS benefit (mOS 21.0 and 14.0 months, HR = 0.79, 95% CI: 0.45 to 1.38). In our study, the PFS advantage of immune combination therapy was also demonstrated, therefore, immune monotherapy should be avoided as much as possible in NSCLC patients receiving first-line immunotherapy.

Previous studies suggested that *KRAS* mutation is mutually exclusive with *EGFR* or *BRAF* mutations in patients with NSCLC 20, 27. However, in this study, we identified a patient with *KRAS* + *BRAF* co-mutation, which suggests that the *KRAS* gene can be mutated simultaneously with other driver genes. This patient had a PFS of 6.4 months, which was lower than 10.1 months for patients with *KRAS* mutation alone. The small number of patients with *ALK*, *MET*, and *RET* mutations included in this study indicates that in the real world, clinicians usually do not consider giving ICIs to this group of patients. This confirms that these patients are difficult to benefit from immunotherapy as suggested in some clinical trials and case reports.

Although patients with higher PD-L1 expression levels benefited more from immunotherapy, there were still patients with negative PD-L1 expression who achieved good PFS in first-line immunotherapy 28, 29. This suggests that PD-L1 negativity is not an absolute contraindication to ICI therapy. Patients with low PD-L1 expression are only less likely to benefit from immunotherapy compared with patients with high PD-L1 expression. However, due to the complexity of immunotherapy mechanisms and the prediction of immunotherapy efficacy by PD-L1 expression status alone, there is still a lack of evidence, and further studies are necessary to determine which patients are more likely to benefit from ICIs.

Inevitably, our study has some limitations. (1) It was a retrospective study, and most of the patient clinical information was obtained from electronic medical records, and errors in the entry of information are an inevitable problem for all retrospective studies. (2) Our study is a single-center study and patient selection may be biased. (3) Few NSCLC patients with driver mutations for which targeted agents are available receive first-line immunotherapy, and we inevitably included fewer patients in our study.

## Conclusion

Our study found that there were differences in immunotherapy efficacy among patients with NSCLC with different driver gene mutation status. KRAS patients could benefit from first-line immunotherapy, with patients with EGFR mutations receiving first-line immunotherapy having a large difference in benefit from standard targeted therapy, and patients with other mutation types having no significant difference in response from mutation-negative patients. Therefore, targeted therapy should be preferred for NSCLC patients with driver gene mutations when targeted agents are available. Second, PD-L1 expression testing should be performed before immunotherapy, and higher PD-L1 predicts better efficacy.

## Figures and Tables

**Table 1:** Demographic characteristics of patients of NSCLC receiving first-line therapy (n = 89).

Characteristic	Positive(n=40)	Negative(n=49)
Sex		
Male	35	41
Female	5	8
Age-yr		
<60	6	14
≥60	34	35
AJCC stage		
III stage	15	20
IV stage	25	29
Histologic type		
Adenocarcinoma	33	23
Squamous cell carcinoma	7	26
Smoking History		
Ever (Current/Former)	32	38
Never	8	11
Treatment mode		
Immune monotherapy	11	10
Combination immunotherapy	29	39
PD-L1 Expression		
Negative (<1%TPS)	15	12
Low (1-49%TPS)	11	13
High (≥50%TPS)	14	24

Abbreviations: PD-L1: programmed cell death-Ligand 1. AJCC: American Joint Committee on Cancer.

**Table 2:** Univariate and multivariate analysis with Progression-free survival in patients.

Variable	Univariate analysis		Multivariate analysis		
	P-value		95% CI		P-value
Sex					
Male	0.616		0.545-2.478		0.697
Female					
Age					
<60	0.13		0.486-1.560		0.641
≥60					
Histologic type					
Squamous cell carcinoma	<0.001		0.591-1.728		0.969
Adenocarcinoma					
AJCC state					
III state	0.753		0.3531.057		0.078
IV state					
Treatment mode					
Immune monotherapy	0.492		0.070-0.864		0.029
Combination immunotherapy					
Smoking History					
Ever (Current/Former)	0.782		0.769-3.056		0.225
Never					
PD-L1 Expression					
Negative (<1%TPS)	0.137		0.362-1.251		0.211
Low (1-49%TPS)					
High (≥50%TPS)					

Abbreviations: PD-L1: programmed cell death-Ligand 1. AJCC: American Joint Committee on Cancer.
